# Scaling up private land conservation to meet recovery goals for grassland birds

**DOI:** 10.1111/cobi.13731

**Published:** 2021-06-30

**Authors:** David C. Pavlacky, Christian A. Hagen, Anne M. Bartuszevige, Rich Iovanna, Thomas Luke George, David E. Naugle

**Affiliations:** ^1^ Bird Conservancy of the Rockies Brighton Colorado USA; ^2^ Department of Fisheries and Wildlife Oregon State University Corvallis Oregon USA; ^3^ Playa Lakes Joint Venture Lafayette Colorado USA; ^4^ Economic and Policy Analysis, Farm Service Agency United States Department of Agriculture Washington D.C. USA; ^5^ Department of Fish, Wildlife, and Conservation Biology Colorado State University Fort Collins Colorado USA; ^6^ W.A. Franke College of Forestry and Conservation University of Montana Missoula Montana USA

**Keywords:** Conservation Reserve Program, Integrated Monitoring in Bird Conservation Regions, Lesser Prairie‐chicken Initiative, population density, population size, U.S. Farm Bill, densidad poblacional, Iniciativa de la Gallina de Pradera Menor, Monitoreo Integrado en las Regiones de Conservación de Aves, Programa de Reservas de Conservación, Proyecto de Ley de Granjas Estadunidense, tamaño poblacional

## Abstract

Long‐term population declines have elevated recovery of grassland avifauna to among the highest conservation priorities in North America. Because most of the Great Plains is privately owned, recovery of grassland bird populations depends on voluntary conservation with strong partnerships between private landowners and resource professionals. Despite large areas enrolled in voluntary practices through U.S. Department of Agriculture's Lesser Prairie‐chicken (*Tympanuchus pallidicinctus*) Initiative (LPCI), the effectiveness of Farm Bill investments for meeting wildlife conservation goals remains an open question. Our objectives were to evaluate extents to which Conservation Reserve Program (CRP) and LPCI‐grazing practices influence population densities of grassland birds; estimate relative contributions of practices to regional bird populations; and evaluate percentages of populations conserved relative to vulnerability of species. We designed a large‐scale impact‐reference study and used the Integrated Monitoring in Bird Conservation Regions program to evaluate bird population targets of the Playa Lakes Joint Venture. We used point transect distance sampling to estimate density and population size for 35 species of grassland birds on private lands enrolled in native or introduced CRP plantings and LPCI‐prescribed grazing. Treatment effects indicated CRP plantings increased densities of three grassland obligates vulnerable to habitat loss, and LPCI grazing increased densities of four species requiring heterogeneity in dense, tall‐grass structure (*α* = 0.1). Population estimates in 2016 indicated the practices conserved breeding habitat for 4.5 million birds (90% CI: 4.0–5.1), and increased population sizes of 16 species , totaling 1.8 million birds (CI: 1.4–2.4). Conservation practices on private land benefited the most vulnerable grassland obligate species (AIC_c_ weight = 0.53). By addressing habitat loss and degradation in agricultural landscapes, conservation on private land provides a solution to declining avifauna of North America and scales up to meet population recovery goals for the most imperiled grassland birds.

## Abstract 2

*Article impact statement*: Voluntary conservation on private land addressing critical habitat needs provides an emerging solution to the decline of grassland birds.

## Abstract (es)

Ampliación de la Conservación en Terrenos Privados para Cumplir los Objetivos de Recuperación para Aves de Pastizales

**Resumen:**

La declinación a largo plazo de las poblaciones ha posicionado a la recuperación de la avifauna de los pastizales entre las prioridades de conservación más importantes en América del Norte. Debido a que la mayor parte de las Grandes Planicies es propiedad privada, la recuperación de las poblaciones de aves de los pastizales depende de la conservación voluntaria sumada a la colaboración entre los terratenientes privados y los profesionales de la gestión de recursos. A pesar de que varias áreas se encuentran inscritas en prácticas voluntarias por medio de la Iniciativa de la Gallina de Pradera Menor (*Tympanuchus pallidicinctus*) (IGPM), la efectividad de la inversión del Proyecto de Ley de Granjas para cumplir con los objetivos de conservación de fauna todavía permanece como una pregunta abierta. Nuestros objetivos se enfocaron en evaluar hasta qué punto el Programa de Reservas de Conservación (PRC) y las prácticas de forrajeo de la IGPM influyen sobre la densidad poblacional de las aves de los pastizales; estimar las contribuciones relativas de las prácticas para las poblaciones de aves regionales; y evaluar el porcentaje de poblaciones conservadas en relación con la vulnerabilidad de la especie. Diseñamos un estudio a gran escala con referencia de impactos y usamos el programa de Monitoreo Integrado en las Regiones de Conservación de Aves para evaluar los objetivos poblacionales de las aves del Proyecto Conjunto de Playa Lakes. Usamos un muestreo de distancia por puntos en transecto para estimar la densidad y el tamaño poblacional de 35 especies de aves de pastizales en los terrenos privados inscritos en plantaciones nativas o introducidas del PRC y en las zonas de forrajeo prescritas por la IGPM. Los efectos del tratamiento indicaron que las plantaciones del PRC incrementaron la densidad de tres especies estrictas de pastizales vulnerables a la pérdida del hábitat, mientras que el forrajeo de la LPCI incrementó la densidad de cuatro especies que requieren heterogeneidad en la estructura de pastos altos y gruesos (*α* = 0.1). Las estimaciones poblacionales indicaron que las prácticas conservaron el hábitat de reproducción para 4.5 millones de aves (90% IC 4.0 ‐ 5.1) e incrementaron el tamaño poblacional de 16 especies, para un total de 1.8 millones de aves (IC 1.4 ‐ 2.4). Las prácticas de conservación en terrenos privados beneficiaron a las especies estrictas de pastizales más vulnerables (peso AIC_c_ = 0.53). Al abordar la pérdida y degradación del hábitat en los paisajes agrícolas, la conservación en terrenos privados proporciona una solución para la avifauna en declinación de América del Norte y se amplía para cumplir con los objetivos de recuperación establecidos para las aves de pastizales que se encuentran en mayor peligro.

## INTRODUCTION

Long‐term population declines have elevated the recovery of grassland avifauna to among the highest conservation priorities in North America (Brennan & Kuvlesky, 2005; Rosenberg et al., 2019). Habitat loss and degradation from intensive agriculture are considered primary threats to populations of grassland birds (Brennan & Kuvlesky, 2005; Vitousek et al., 1997). Understanding threats and vulnerability are important in conservation planning to set objectives and ensure the most pressing conservation problems are solved (Rosenberg et al., 2019). In anthropogenically altered landscapes, active management may be necessary to restore historical ecosystem processes and stabilize declining populations (Vickery & Herkert, 1999). Understanding the relative effectiveness of alternative actions to achieve population objectives is crucial for tangible conservation outcomes (Lyons et al., 2008) and addressing commonly held presumptions that actions will produce successful outcomes and mitigate threats (Wilson et al., 2005).

Because most of the Great Plains is privately owned, species recovery ultimately depends on conservation initiatives with strong partnerships between private landowners and resource professionals (Drum et al., 2015). Habitat management for imperiled prairie grouse, such as Lesser Prairie‐chicken (*Tympanuchus pallidicinctus*), may provide the social and economic wherewithal for the large‐scale conservation of grassland birds on privately owned working lands (Brennan & Kuvlesky, 2005). The U.S. Farm Bill incentivizes conservation on privately owned lands by providing cost‐share payments to implement U.S. Department of Agriculture (USDA) conservation practices (Briske et al., 2017). The Conservation Reserve Program (CRP) and Lesser Prairie‐chicken Initiative (LPCI) include conservation practices for managing grassland habitat for Lesser Prairie‐chicken (Hagen et al., 2016). Practices are implemented within the Playa Lakes Joint Venture (PLJV, 2007) regional partnership to connect private agricultural producers with practices to achieve bird conservation objectives in the southern Great Plains (Brennan & Kuvlesky, 2005). The CRP practices provides financial incentives for private producers to take cropland out of production and restore perennial grassland. Although CRP plantings were originally designed to reduce cultivation on marginal or vulnerable lands and address soil erosion, the objectives have expanded to include wildlife concerns (Hellerstein, 2017). The CRP shows promise for recovering grassland bird populations in landscapes affected by grassland loss (Herkert, 2009). The LPCI‐prescribed grazing practice involves cost‐sharing for conservative grazing systems (i.e., includes deferred grazing) to promote residual cover of perennial grasses and forbs to improve habitat for Lesser Prairie‐chicken (Van Pelt et al., 2013). Prescribed grazing is useful for managing grassland degradation and restoring structural heterogeneity essential to avian biodiversity (Derner et al., 2009). Effectiveness monitoring provides a platform for evidence‐based conservation on private lands (Briske et al., 2017), with treatment effects scaling up to predict contributions of local management to regional bird populations.

Our objectives were to evaluate extents to which CRP‐restoration and LPCI‐grazing practices on private land influence population densities of grassland birds, estimate relative contributions of conservation practices to regional bird populations, and evaluate percentages of populations conserved relative to Partners in Flight (2019) breeding‐season vulnerability. We evaluated a priori hypotheses for each objective. We hypothesized grassland obligates (Vickery & Herkert, 1999; Appendix S1) threatened by habitat loss have higher densities on CRP lands relative to agricultural reference lands (Herkert, 2009). We predicted native CRP would primarily benefit grassland obligates, whereas introduced CRP grassland would benefit facultative species (Vickery & Herkert, 1999; Thompson et al., 2009). In addition, we predicted species vulnerable to habitat degradation would benefit from heterogeneity in dense, tall‐grass structure produced by LPCI grazing (Hovick et al., 2015; Lipsey & Naugle, 2017). Because life‐history traits underlie increasing or decreasing abundance in modified agricultural landscapes (McGill et al., 2015), we evaluated predictions for positive or negative effects of conservation based on species’ habitat requirements (Appendix S2). We hypothesized treatment effects scale up to meet population recovery objectives for priority species in the PLJV (2007) and that the CRP restoration‐ and LPCI‐grazing practices make the largest contributions to populations of grassland species with high vulnerability to habitat loss and degradation (Appendix S1).

## METHODS

### Study area

**FIGURE 1:**
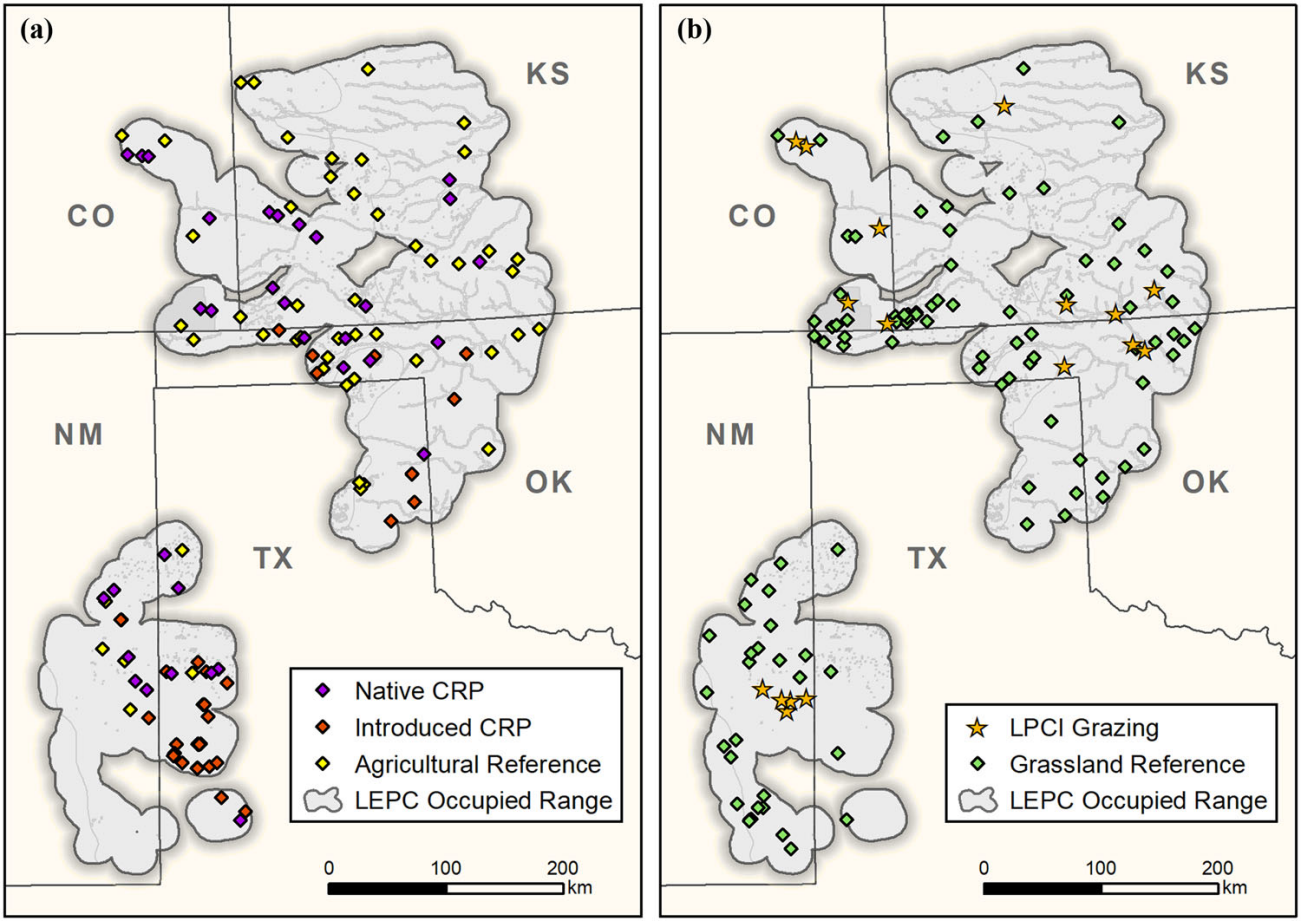
Approximate location of (a) native and introduced Conservation Reserve Program (CRP) plantings and reference agricultural lands and (b) Lesser Prairie‐chicken Initiative (LPCI)‐prescribed grazing and reference grasslands in the occupied range of the Lesser Prairie‐chicken (LEPC) (CO, Colorado; KS, Kansas; NM, New Mexico; OK, Oklahoma; TX, Texas), 2015–2017

The study area was the occupied range of Lesser Prairie‐chicken plus a 16‐km area outside their range (161,761 km^2^) in Colorado, Kansas, Oklahoma, New Mexico, and Texas (USA) (Figure 1). The study area was in the Shortgrass Prairie and Central Mixed‐grass Prairie Bird Conservation Regions (BCR 18 and 19, respectively); a small portion was in the Chihuahua Desert (BCR 35) (BSC & NABCI, 2014).

We evaluated two CRP practices for restoring perennial grassland in former cropland: seed mixes for introduced grasses and legumes (introduced CRP plantings) and native grasses (native CRP plantings). We assumed typical 10‐year contracts and similar age of active native ($\underline{x}$ = 3.6 year [SD 0.9]) and introduced planting ($\underline{x}$ = 4.7 year [SD 2.4]) in CRP fields, but because the number of re‐enrollment contracts was unknown, these represented minimum field ages. Longitudinal distribution of practices was similar, but native plantings ($\underline{x}$ = 35.2° [SD 1.8]) occurred at slightly lower latitudes than introduced plantings ($\underline{x}$ = 36.4° [SD 1.7]). From a survey of plant species composition (Ripper et al., 2008), introduced CRP plantings were dominated by two non‐native warm‐season grasses: weeping lovegrass (*Eragrostis curvula*) and old world bluestem (*Bothriochloa ischaemum*). Native CRP plantings were characterized by native warm‐season grasses, such as sideoats grama (*Bouteloua curtipendula*), blue grama (*Bouteloua gracilis*), and cool‐season western wheatgrass (*Pascopyrum smithii*).

The LPCI‐grazing practice involves managing stocking rates, rotations, and grazing intensity and duration to meet nesting and brood‐rearing habitat requirements of Lesser Prairie‐chicken (Van Pelt et al., 2013). Common plant species included blue grama, buffalograss (*Bouteloua dactyloides*), and sand bluestem (*Andropogon hallii*) interspersed with sand sagebrush (*Artemisia filifolia*) or sand shinnery oak (*Quercus havardii*). Recommendations for grazing management in the northern range (Figure 1) included maintaining nesting cover with plant height along west‐east gradients >25 to >40 cm, plant foliar cover >60%, and sand sagebrush canopy cover approximately 15% (Hagen et al., 2013). Recommendations in the southern range (Figure 1) included maintaining plant height along west‐east gradients >36 to >50 cm, plant foliar cover >35%, and sand shinnery oak canopy cover approximately 20% (Hagen et al., 2013).

### Sampling design

**FIGURE 2:**
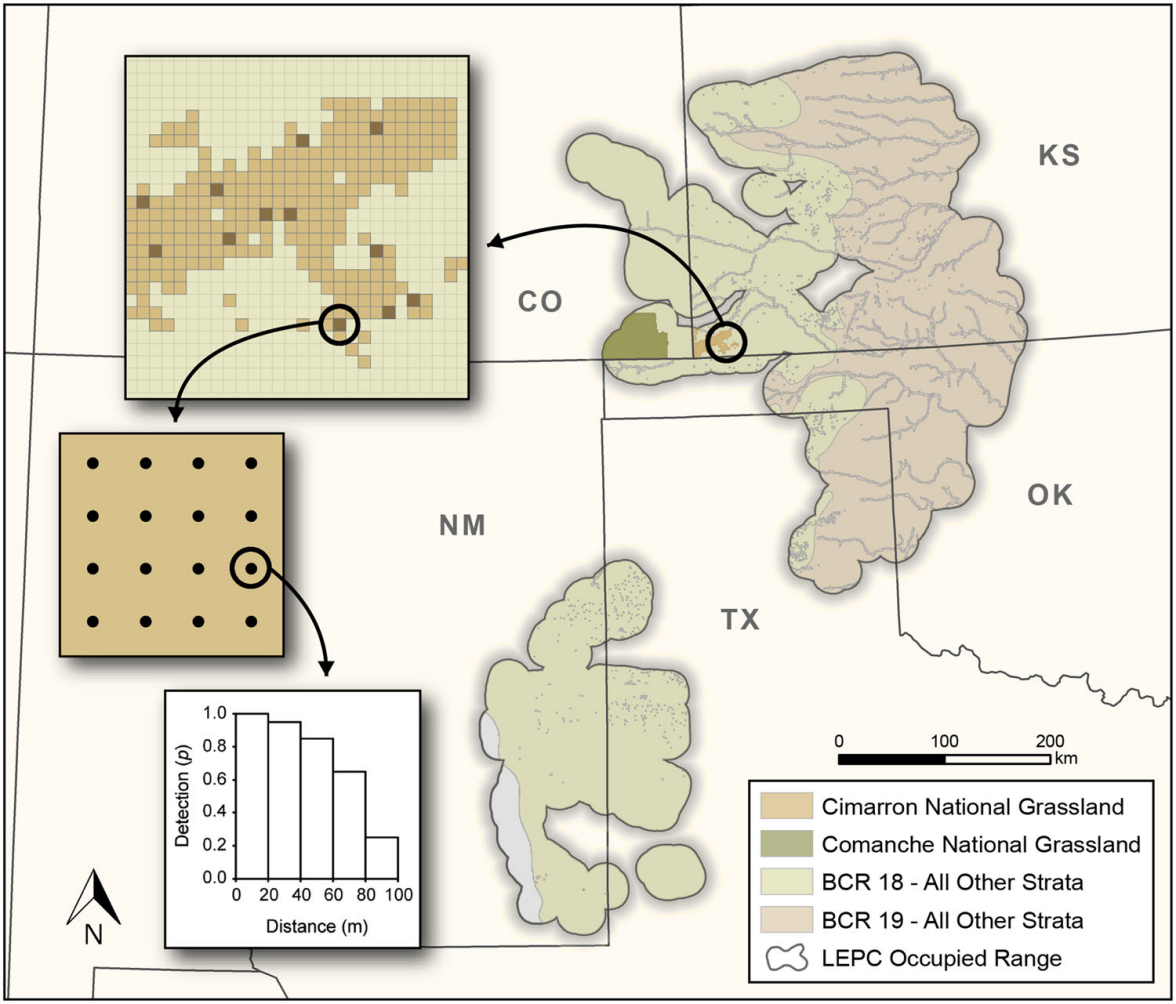
The hierarchical sampling design of the Integrated Monitoring in Bird Conservation Regions program in the occupied range of the Lesser Prairie‐chicken (LEPC), Colorado (CO), Kansas (KS), New Mexico (NM), Oklahoma (OK), and Texas (TX), 2015–2017 (colored regions and gray boundaries, state by Bird‐Conservation‐Region [BCR] strata from the monitoring program)

We designed a large‐scale impact‐reference study (Morrison et al., 2008) based on an alternate and separate stratification scheme for the Integrated Monitoring in Bird Conservation Regions (IMBCR) program (Pavlacky et al., 2017) (Figures 1 and 2). The design represents an observational, or mensurative, study with replicated and randomized sampling of treatments and matching reference (control) groups representing purported treatment contrasts over space (Eberhardt & Thomas, 1991). Treatment strata included lands enrolled in introduced and native CRP plantings and LPCI grazing. Treatment strata overlapped baseline IMBCR strata, but strata were appropriately held separate in the stratified analysis. Reference landscapes were developed by poststratifying IMBCR data based on vegetation measured at point‐count plots. Sampling units were 1‐km^2^ grid cells containing 16 unlimited‐radius point‐count plots (Figure 2). We monitored breeding‐season abundance of adult birds with 6‐min point counts from April 20 to June 15 conducted from 0.5 h before sunrise to 5 h after sunrise (Appendix S3), in compliance with Guidelines to the Use of Wild Birds in Research (Fair et al., 2010). Not all landowners granted permission within every sampling unit, often resulting in <16 plots per unit.

### Conservation reserve program

We developed sampling frames for CRP treatments by intersecting the 1 × 1 km U.S. National Grid (USNG) with 2015 Common Land Unit geospatial data (USDA, 2014) within a Geographic Information System (GIS) (ArcGIS Version 10.1). We stratified sampling frames according to grid cells with ≥40% land cover of native or introduced CRP plantings and by BCR 18 and 19, resulting in four strata. As of 2015, 13,718 km^2^ of land area was enrolled in native CRP and 1425 km^2^ was enrolled in introduced CRP.

We selected a spatially balanced sample of 1200 grid cells with Generalized Random Tessellation Stratification (GRTS) (Stevens & Olsen, 2004). In partnership with USDA, Farm Service Agency (FSA), we mailed 1430 landowner information return cards to producers within the samples requesting permission to access CRP lands, and of these, 105 producers granted permission. In 2016, we selected a sample of 33 grid cells (322 point‐count plots) for introduced CRP plantings and 33 grid cells (293 point‐count plots) for native CRP plantings in proportion to strata area (Figure 1). Samples of introduced plantings ($\underline{x}$ = 34.2° [SD 1.4]) occurred at lower latitudes than native plantings ($\underline{x}$ = 36.4° [SD 1.8]) (Figure 1). Herbaceous ground cover measured at point‐count plots was similar for introduced ($\underline{x}$ = 20.9% [SD 9.3]) and native ($\underline{x}$ = 22.8% [SD 9.7]) plantings, and residual grass height was similar for introduced ($\underline{x}$ = 36.7 cm [SD 13.4]) and native ($\underline{x}$ = 37.4 cm [SD 13.1]) plantings. Power analyses indicated a sample size of 35 grid cells in each stratum would detect a 30% difference in point occupancy.

We developed agricultural reference contrasts by poststratifying point‐count plots according to cropland and rural vegetation composed of seminatural vegetation along fence rows, ditches, center pivot corners, and pastures (Figure 1) (Pavlacky et al., 2017). We sampled 66 grid cells (349 point‐count plots) on agricultural reference lands from 2015 through 2017.

### LPCI‐prescribed grazing

We recruited 17 producers participating in LPCI grazing in coordination with USDA, Natural Resources Conservation Service (NRCS) (Figure 1). We developed a stratified sampling frame by intersecting the USNG with treatment boundaries for 17 LPCI ranches. We stratified the sampling frame by four ecoregions from the Lesser Prairie‐chicken range‐wide conservation plan (Van Pelt et al., 2013). Because a typical LPCI‐grazing contract is 5 years, we calculated areas of active LPCI grazing as running totals for the previous 5 years (1887 km^2^ as of 2016). We used GRTS (Stevens & Olsen, 2004) to select a sample of 30 grid cells per year from 2015 through 2017 (Figure 1; 91 grid cells, 1074 point‐count plots). Residual grass height ($\underline{x}$ = 42.8 cm [SD 7.9]) and foliar plant cover ($\underline{x}$ = 37.0% [SD 5.5]) measured at point‐count plots were consistent with recommended vegetation structure, but shrub cover ($\underline{x}$ = 10.7% [SD 4.1]) was lower than recommended (Hagen et al., 2013).

We developed grassland reference contrasts, representing average grazing conditions by poststratifying point‐count plots according to grassland and shrubland vegetation types (Pavlacky et al., 2017). From 2015 through 2017, we selected a spatially balanced sample (Stevens & Olsen, 2004) of 139 grid cells (1187 point‐count plots) on reference grasslands (Figure 1). Foliar plant cover ($\underline{x}$ = 38.2% [SD 6.3]) was similar to LPCI grazing, whereas residual grass height ($\underline{x}$ = 31.4 cm [SD 4.6]) and shrub cover ($\underline{x}$ = 4.2% [SD 2.8]) were marginally lower than the practice.

### Statistical analyses

We evaluated hypotheses for conservation practices with combined design‐ and model‐based inference (Williams & Brown, 2019). We investigated treatment effects for 35 species of grassland birds, including 11 grassland obligates and 24 grassland facultative species (Vickery & Herkert, 1999) (Appendix S1). For each species, we estimated density and population size separately for treatment, reference, and study‐area strata with point‐transect distance sampling (Buckland et al., 2001) (Figure 2). We fitted conventional and multiple covariate distance sampling models with package mrds (Version 2.2.0) in the R statistical computing environment (version 3.4.3) (Appendix S3).

We estimated mean population density for treatment and reference strata with a stratified random estimator weighted by area for each stratum and year $\hat{D}=\sum_{i=1}^{n}\sum_{j=1}^{t}w_{ij}{\hat{d}}_{ij}$, where $\hat{D}$ is aggregated density, *n* is the number of strata, *t* is the number of years, $w_{ij}$ is proportional areas of stratum *i* and year *j* ($\sum_{i=1}^{n}\sum_{j=1}^{t}w_{ij}=1)$, and ${\hat{d}}_{ij}$ is density for stratum *i* and year *j* (Pavlacky et al., 2017). We estimated mean overall density (${\hat{D}}_{\mathrm{tot}}$) by applying the above estimator to estimates of density weighted by the proportional areas of 26 strata intersecting the study area in 2016 and 2017 from the IMBCR program (Pavlacky et al., 2017) (Figure 2).

We calculated treatment effects for each species ($\hat{\Delta }$) according to $\hat{\Delta }\;=\;{\hat{D}}_{\mathrm{trt}}\;-\;{\hat{D}}_{\mathrm{ref}}$, where ${\hat{D}}_{\mathrm{trt}}$ and ${\hat{D}}_{\mathrm{ref}}$ are estimated population densities for treatment and reference strata, respectively.

We estimated relative population change for each species (${\hat{N}}_{\mathrm{rel}}$) according to ${\hat{N}}_{\mathrm{rel}}=\sum_{k=1}^{3}{\hat{\Delta }}_{k}A_{k}$, where ${\hat{\Delta }}_{k}$ is the density treatment effect and $A_{k}$ is the 2016 regional area for conservation practice *k*. In addition, we estimated absolute population size (${\hat{N}}_{\mathrm{abs}}$) according to ${\hat{N}}_{\mathrm{abs}}=\sum_{k=1}^{3}{\hat{D}}_{k}A_{k}$, where ${\hat{D}}_{k}$ is the mean density and $A_{k}$ is the 2016 regional area for practice *k*. We estimated cumulative contributions of conservation to avian populations by summing population sizes of 35 species. We estimated regional population size according to ${\hat{N}}_{\mathrm{tot}}={\hat{D}}_{\mathrm{tot}}A_{\mathrm{tot}}$, where ${\hat{D}}_{\mathrm{tot}}$ is the mean density and $A_{\mathrm{tot}}$ is the area of the study area (Buckland et al., 2001). We estimated percent relative change from treatment effects (${\hat{\delta }}_{\mathrm{rel}}$) and absolute percentage of population conserved (${\hat{\delta }}_{\mathrm{abs}}$) according to $\hat{\delta }\;=\;\left(\hat{N}/{\hat{N}}_{\mathrm{tot}}\right)\;\times \;100$, where $\hat{N}$ is the relative or absolute population size from overall conservation and ${\hat{N}}_{\mathrm{tot}}$ is the regional population size. We approximated SE for densities, treatment effects, and population sizes with the delta method (Powell, 2007), calculated Satterthwaite 90% confidence intervals (CIs) for estimated effects (Buckland et al., 2001), and evaluated statistical support (*α* = 0.1) for treatment effects based on CIs relative to zero. Relative change associated with treatment effects (${\hat{\delta }}_{\mathrm{rel}}$) is a spatial trend measured by the ratio of abundance for treatment and reference lands at two different places (Yoccoz et al., 2001). We determined evidence of meeting regional population targets by evaluating CIs for relative percent change over space with respect to trends for percent change over time used to set PLJV (2007) population objectives, as well as Breeding Bird Survey (BBS) trends for percent change in the region (Sauer et al., 2017) (Appendix S1).

We used a generalized linear model (R function glm) to evaluate the hypothesis that percentage of populations conserved increases along a gradient of breeding‐season vulnerability. We fitted models with a Gaussian family distribution and identity link function and weighted the response variable by (1 / coefficient of variation) to account for variability in precision. We developed a continuous vulnerability covariate based on mean Partners in Flight (2019) regional combined breeding‐season scores weighted by areas of BCR 18, 19, and 35 in the study area (Appendix S1). We log*_e_* transformed vulnerability scores to allow nonlinear covariate relationships. We evaluated strength of evidence for hypotheses with information‐theoretic model selection (Burnham & Anderson, 2002).

## Results

### Conservation practices and population density

Population densities of several species vulnerable to habitat loss increased in native and introduced CRP plantings, which supported our prediction. Densities of obligate Cassin's Sparrow (*Peucaea cassinii*), Grasshopper Sparrow (*Ammodramus savannarum*), and Eastern Meadowlark (*Sturnella magna*) were greater on CRP plantings than reference agricultural lands (Table 1; Appendices S2 and S4). Density of Grasshopper Sparrow was greater on native CRP plantings, but opposite to predictions, densities of Cassin's Sparrow and Eastern Meadowlark were greater on introduced plantings. As predicted, densities of several facultative species were greater on introduced than native CRP plantings (e.g., Mourning Dove [*Zenaida macroura*], American Kestrel [*Falco sparverius*], and Western Kingbird [*Tyrannus verticalis*]). However, positive effects of introduced CRP for Northern Bobwhite (*Colinus virginianus*) and Scaled Quail (*Callipepla squamata*) were opposite to predictions. Facultative species predicted to increase in agricultural landscapes showed lower densities on CRP plantings than agricultural reference lands, including Ring‐necked Pheasant (*Phasianus colchicus*), Killdeer (*Charadrius vociferus*), Red‐winged Blackbird (*Agelaius phoeniceus*), and Brown‐headed Cowbird (*Molothrus ater*).

**TABLE 1:** Estimated treatment effects for population density (km^−2^) and lower (LCL) and upper (UCL) 90% confidence limits for prescribed grazing of the Lesser Prairie‐chicken Initiative and for native and introduced Conservation Reserve Program (CRP) plantings relative to reference grassland (2016) or agricultural land from the impact‐reference design (2015–2017)

	Prescribed grazing			Native CRP plantings			Introduced CRP plantings		
Species	Effect	LCL	UCL	Effect	LCL	UCL	Effect	LCL	UCL
Northern Bobwhite^b^	2.05^a^	0.85	3.25	–0.10	–2.22	2.02	5.36^a^	2.80	7.93
Scaled Quail^b^	–1.18^a^	–2.36	0.00	1.00	–0.10	2.11	1.87^a^	0.61	3.14
Ring‐necked Pheasant	0.03	–0.19	0.25	–2.71^a^	–3.47	–1.95	–3.45^a^	–4.12	–2.78
Mourning Dove	2.89^a^	1.32	4.45	2.90^a^	0.05	5.74	10.10^a^	6.64	13.57
Common Nighthawk^d^	0.76^a^	0.23	1.28	–1.49^a^	–2.74	–0.24	–1.23	–2.53	0.07
Killdeer^d^	0.04	–0.74	0.81	–5.57^a^	–7.10	–4.04	–5.30^a^	–6.81	–3.79
Long‐billed Curlew^b^, ^c^, ^d^	–0.11	–0.38	0.17	–0.03	–0.13	0.08	–0.06	–0.15	0.02
Turkey Vulture	1.40^a^	0.21	2.60	–0.19	–0.44	0.06	–0.14	–0.40	0.12
Swainson's Hawk^b^, ^c^	0.05	–0.02	0.11	–0.07	–0.25	0.11	–0.04	–0.24	0.15
Burrowing Owl^b^, ^c^, ^d^	–0.11	–0.45	0.23	–	–	–	–	–	–
American Kestrel	0.02	–0.12	0.16	–0.02	–0.10	0.06	0.38^a^	0.00	0.75
Ash‐throated Flycatcher	–0.14	–0.37	0.09	0.00	–0.01	0.01	0.80	–0.22	1.82
Western Kingbird	–1.42	–3.40	0.56	–2.51	–5.61	0.60	6.50^a^	1.58	11.42
Eastern Kingbird	3.11^a^	1.38	4.84	–	–	–	–	–	–
Scissor‐tailed Flycatcher	–0.75	–1.82	0.32	–0.25	–1.54	1.04	0.71	–0.43	1.84
Say's Phoebe	0.00	–0.07	0.08	–	–	–	–	–	–
Loggerhead Shrike^b^	0.05	–0.09	0.18	0.07	–0.06	0.20	0.20	–0.05	0.44
Chihuahuan Raven^b^, ^d^	–0.12	–0.37	0.14	0.11	–0.10	0.31	0.23	–0.08	0.55
Horned Lark^c^, ^d^	–28.64^a^	–38.88	–18.41	–30.37	–51.91^a^	–8.82	–54.91^a^	–73.86	–35.97
Cassin's Sparrow^b^, ^c^	13.37^a^	4.73	22.01	23.80^a^	16.44	31.16	45.20^a^	36.74	53.65
Grasshopper Sparrow^b^, ^c^, ^d^	–19.54^a^	–32.39	–6.69	70.99^a^	45.76	96.22	38.34^a^	13.37	63.31
Lark Sparrow^b^, ^d^	–1.77	–8.40	4.86	0.01	–2.58	2.61	3.31	–0.32	6.93
Lark Bunting^b^, ^c^, ^d^	–5.45	–11.40	0.51	12.87	–5.95	31.69	–8.51	–18.29	1.28
Field Sparrow	1.18^a^	0.53	1.84	–	–	–	–	–	–
Eastern Meadowlark^b^, ^c^	11.78^a^	8.25	15.31	8.42^a^	1.54	15.31	20.45^a^	14.57	26.33
Western Meadowlark^b^, ^c^, ^d^	–9.98^a^	–13.30	–6.66	–1.50	–7.05	4.04	–8.11^a^	–14.36	–1.86
Red‐winged Blackbird^d^	0.17	–2.41	2.76	–31.20^a^	–40.19	–22.20	–27.64^a^	–37.28	–18.00
Brown‐headed Cowbird^d^	0.70	–2.47	3.87	–24.20^a^	–31.12	–17.28	–21.78^a^	–28.87	–14.70
Dickcissel^c^, ^d^	2.51	–3.01	8.03	–23.37^a^	–32.60	–14.14	–24.91^a^	–33.92	–15.90

^a^ Measurable effects sizes (*α* = 0.1).

^b^ Partners in Flight breeding‐season vulnerability (>0.5 quantile).

^c^ Grassland obligate species.

^d^ Species requiring moderate to heavy grazing or short grass conditions.

We found support for the hypothesis that species requiring heterogeneity in tall‐grass structure benefited from LPCI grazing. As predicted, densities of Northern Bobwhite, Cassin's Sparrow, Field Sparrow (*Spizella pusilla*), and Eastern Meadowlark were greater for LPCI grazing than reference grasslands (Table 1; Appendices S2 and S4). However, we found little evidence of treatment effects for Lark Bunting (*Calamospiza melanocorys*) and Dickcissel (*Spiza americana*), and patterns for Scaled Quail and Grasshopper Sparrow were opposite predictions. As predicted, two grassland obligates favoring short‐grass conditions, Horned Lark and Western Meadowlark (*Sturnella neglecta*), exhibited lower densities for LPCI grazing than reference grasslands.

### Contributions of private land conservation to regional bird populations

**FIGURE 3:**
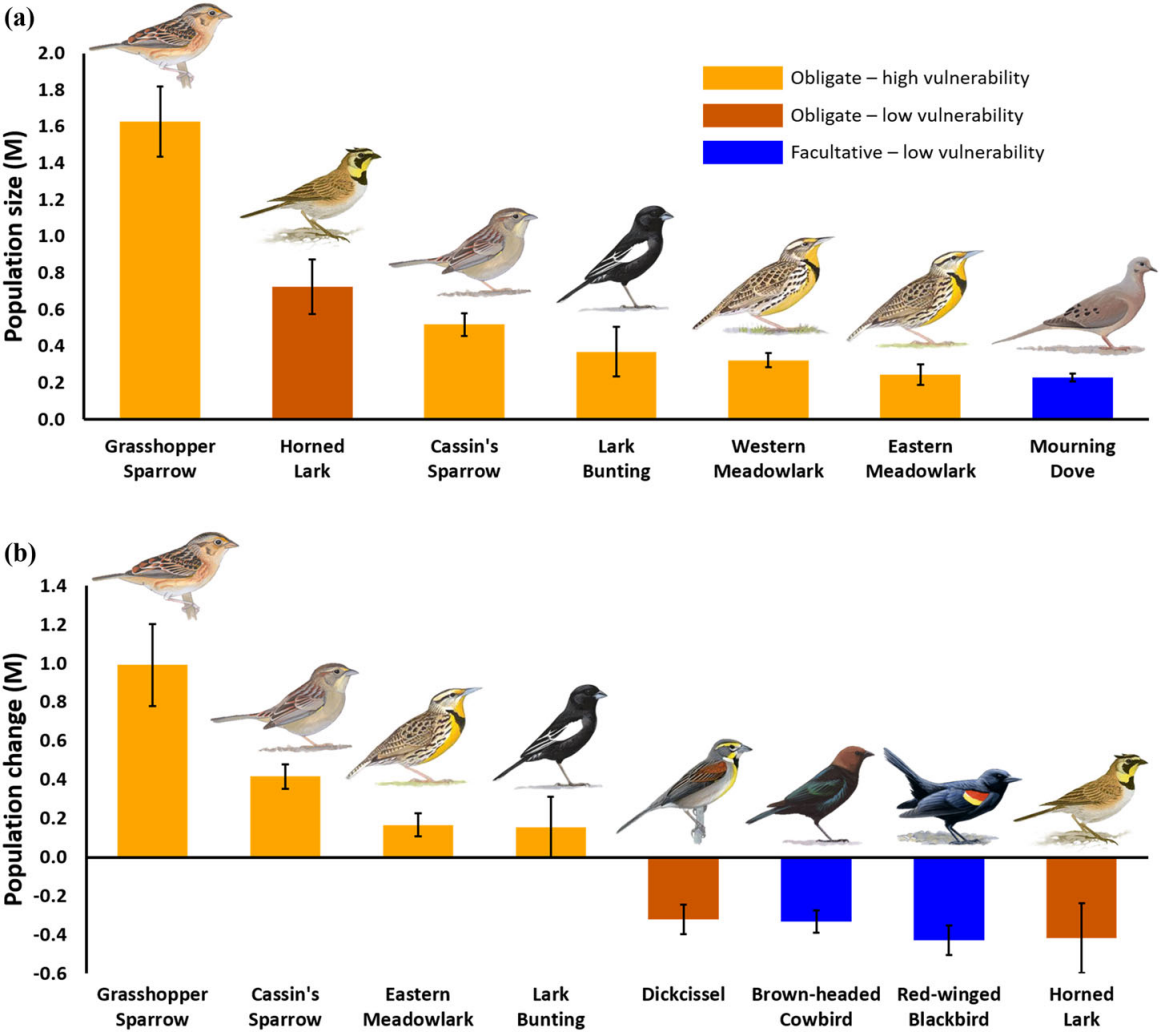
Estimated overall (a) population size for land enrolled in conservation practices and (b) population change from treatment effects relative to reference strata for grassland birds by grassland specialization and Partners in Flight breeding‐season vulnerability in Colorado, Kansas, New Mexico, Oklahoma, and Texas, 2016 (error bars, SE)

Conservation practices implemented in 2016 supported large populations (${\hat{N}}_{\mathrm{abs}}$) of Grasshopper Sparrow, Cassin's Sparrow, and Eastern Meadowlark (Figure 3; Appendix 5). Percentage of populations conserved (${\hat{\delta }}_{\mathrm{abs}}$) exceeded the 10.5% regional availability of practices for Eastern Meadowlark (25.8%, 90% CI: 15.1–38.1), Grasshopper Sparrow (21.9%, CI: 16.4–28.1), and Cassin's Sparrow (19.2%, CI: 14.2–24.8) (Appendix S6). Cumulative population size across all 35 species occurring on practices was 4.5 M (90% CI: 4.0–5.1) (Appendix S5).

Species with greatest relative population changes (${\hat{N}}_{\mathrm{rel}}$) were grassland obligates with high vulnerability to habitat loss from agricultural conversion and habitat degradation from overgrazing, including Cassin's Sparrow, Grasshopper Sparrow, Eastern Meadowlark, and Lark Bunting (Figure 3a; Appendix S5). Percent change (${\hat{\delta }}_{\mathrm{rel}}$) for Cassin's Sparrow (15.4%, 90% CI: 10.9–20.6) indicated population size was greater than the 10.5% regional availability of practices. Percent change for 10 species with CIs covering 10.5% indicated contributions to populations were in proportion to availability of practices (Appendix S6). Percent change was positive for seven species, but CIs excluding 10.5% indicated contributions to populations were less than availability of practices (Appendix S6). Percent population changes from overall conservation met (CIs covered target) or exceeded (CIs above target) percent annual trend objectives for eight priority species in the PLJV (2007) (Appendix S6): Northern Bobwhite, Scaled Quail, Loggerhead Shrike (*Lanius ludovicianus*), Cassin's Sparrow, Grasshopper Sparrow, Lark Sparrow (*Chondestes grammacus*), Lark Bunting, and Eastern Meadowlark. Estimates of relative change were greater than (CIs above trend) or not different from BBS trend estimates (CIs covered trend) for nine of 11 declining species (Appendices S1 and S6). Cumulative population change from overall conservation for 16 species with positive effects was 1.8 M (90% CI: 1.4–2.4) (Appendix S5).

Species with the greatest negative population changes (${\hat{N}}_{\mathrm{rel}}$) were species with low vulnerability to habitat loss from agricultural conversion and habitat degradation from over grazing, including obligate Horned Lark (cultivated land) and Dickcissel (patchy grassland) and facultative Red‐winged Blackbird and Brown‐headed Cowbird (Figure 3b; Appendices S2 and S5). No species showed negative relative changes (${\hat{\delta }}_{\mathrm{rel}}$) below 10.5% regional availability of conservation practices (Appendix S6). Negative percent changes for seven species with CIs including −10.5% indicated effects were proportional to availability of practices (Appendix S6). We found evidence of negative percent change for additional five species, but CIs excluding −10.5% indicated changes were proportionally less than availability of practices (Appendix S6). Conservation practices were unable to meet PLJV (2007) population objectives for seven priority species (Appendix S6): Ring‐necked Pheasant, Long‐billed Curlew (*Numenius americanus*), Swainson's Hawk (*Buteo swainsoni*), Burrowing Owl (*Athene cunicularia*), Western Kingbird, Scissor‐tailed Flycatcher (*Tyrannus forficatus*), and Dickcissel. Cumulative population change from overall conservation for 14 species with negative effects was −1.9 M (90% CI: −2.4 to −1.6) (Appendix S5).

### Species vulnerability and contributions to regional populations

**FIGURE 4:**
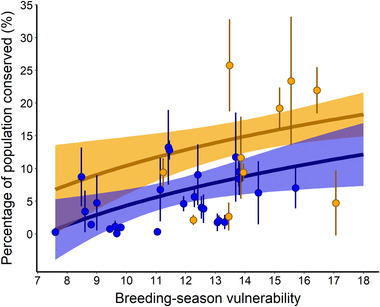
Overall percentage of populations conserved by the Conservation Reserve Program practices and Lesser Prairie‐chicken Initiative grazing as a function of grassland specialization and Partners in Flight breeding‐season vulnerability in Colorado, Kansas, New Mexico, Oklahoma, and Texas, 2016 (points, percentage of populations conserved by conservation practices for 35 bird species within the study area [1 SE]; bold trend lines, predicted contributions to population size; ribbons, 95% confidence intervals)

The best model explaining percentage of populations conserved included additive effects of breeding‐season vulnerability and grassland specialization (AIC_c_ weight = 0.53) (Appendix S7), supporting the hypotheses that most vulnerable and specialized species benefit from the practices. Percentage of populations conserved increased as log*_e_* breeding‐season vulnerability increased ($\hat{\beta }$ = 13.3, 90% CI: 3.4–23.2) and was greater for grassland obligates than facultative species ($\hat{\beta }$ = 6.1, CI: 2.2–10.0) (Figure 4).

## DISCUSSION

Outcomes of USDA Farm Bill assessments (Briske et al., 2017) indicated CRP and LPCI grazing provided habitat restoration and improvement necessary for several imperiled grassland birds (Rosenberg et al., 2019) to reach population recovery goals in the southern Great Plains (PLJV, 2007). Considering the number of grassland birds in North America declined by 718 million over the last 50 years (Rosenberg et al., 2019), these practices conserved breeding habitat for 4.5 million grassland birds per year over a 162,000‐km^2^ agricultural landscape. Comprehensive study of 35 bird species indicated greatest beneficiaries of practices were grassland obligates with high vulnerability to habitat loss from agricultural conversion and degradation from overgrazing (Brennan & Kuvlesky, 2005; Herkert, 2009). Vulnerable species not conserved by CRP or LPCI grazing may benefit from increasing intensity of rotational grazing systems (Derner et al., 2009) and transitioning expiring CRP plantings to working rangelands with additional structural heterogeneity from fire or grazing disturbance.

The CRP‐restoration practices in the region were primarily designed to manage populations of Lesser Prairie‐chicken with high vulnerability to habitat loss from agricultural conversion (Hagen et al., 2016). Implementation of CRP has evolved over time and increasingly provides incentives for private producers to plant native CRP seed mixes to benefit wildlife (Thompson et al., 2009), including Lesser Prairie‐chicken (Van Pelt et al., 2013), grassland obligates and other species of conservation concern (Brennan & Kuvlesky, 2005). We found several facultative grassland species were more abundant on introduced than native CRP plantings, supporting the prediction that introduced CRP was less important for grassland obligates (Thompson et al., 2009). However, high densities of Northern Bobwhite and Scaled Quail on introduced CRP plantings were counter to predictions, and although introduced bunchgrass may provide nesting cover, native grasses are better habitat for prairie quail (Brennan & Kuvlesky, 2005). Otherwise, our results were largely consistent with the findings of large‐scale evaluations of CRP (Herkert, 2009; Thompson et al., 2009). Despite requirements of midcontract management and recent emphasis on burning and grazing practices to improve Lesser Prairie‐chicken habitat (Van Pelt et al., 2013), our results were similar to other studies indicating current frequency and intensity of midcontract management may be insufficient for species requiring greater habitat heterogeneity, including Northern Bobwhite, Ring‐necked Pheasant, Common Nighthawk, Long‐billed Curlew, Killdeer, and Horned Lark (Derner et al., 2009).

The conservative LPCI‐grazing practice was designed to promote vegetation composition and structure (i.e., residual cover of perennial grasses and forbs) to improve breeding habitat for Lesser Prairie‐chicken (Hagen et al., 2013). We interpreted LPCI‐grazing treatment effects in terms of providing heterogeneity in grassland structure (i.e., tall, dense grass) (Hovick et al., 2015; Lipsey & Naugle, 2017) relative to average rangeland conditions in the region with continuous livestock grazing and reduced heterogeneity (Derner et al., 2009). The overall negative effects of LPCI grazing on Grasshopper Sparrow and Western Meadowlark and lack of effects for Lark Bunting and Dickcissel may be complicated because these species typically show positive responses to moderate grazing in mixed‐grass prairie and negative responses to heavy grazing in shortgrass prairie (Bock et al., 1993). Generally, we found large positive effects of LPCI grazing for species intolerant of high grazing pressure and negative effects for species benefiting from intensive grazing (Bock et al., 1993; Appendix S2), suggesting the conservative LPCI‐grazing practice may lack sufficient heterogeneity in short‐grass conditions required by species adapted to historical grazing disturbance (Derner et al., 2009).

Overall, cumulative relative population changes from conservation practices were equally split between winners (1.8 M) and losers (−1.9 M), largely reflecting propensity of species to decrease or increase in modified agricultural landscapes (McGill et al., 2015). Species with the largest positive population changes were grassland obligates with high vulnerability to habitat loss from agricultural conversion and habitat degradation from over grazing, including Cassin's Sparrow, Grasshopper Sparrow, Lark Bunting, and Eastern Meadowlark (Bock et al., 1993; Vickery & Herkert, 1999). In contrast, species with the largest negative population changes are increasers in modified agricultural landscapes and either show mixed effects or increases with heavy grazing pressure (Bock et al., 1993), including Horned Lark, Red‐winged Blackbird, Brown‐headed Cowbird, Dickcissel, and Killdeer (Billerman et al., 2020) (Appendix S2). Despite affinity for highly modified agricultural landscapes, Horned Lark and Killdeer are declining in the region (Appendix S1), indicating additional conservation practices are needed for species requiring heterogeneity in bare‐ground and short‐grass conditions from intensive grazing, fire, or prairie dog (*Cynomys* spp.) disturbance (Derner et al., 2009; Hovick et al., 2015).

The main limitations of our study involve limited causal inference from impact‐reference designs and temporal uncertainty inherent to highly variable grassland ecosystems. Estimation of treatment effects from quasi‐experimental designs, such as before‐after‐control‐impact and impact‐reference, often shows bias relative to true experiments with random assignment of treatments to experimental units (Adams et al., 2019). Using counterfactual reasoning, impact‐reference treatments assumed constant selection of initial treatments and constant outcomes over space and time (Adams et al., 2019). Large positive effects of introduced CRP relative to agricultural lands for Northern Bobwhite, Scaled Quail, Cassin's Sparrow, and Eastern Meadowlark may partially reflect selection bias from correlation between southern distribution of species (Billerman et al., 2020) and introduced CRP (Figure 1). Although design‐based inference provides robust inference to density and population size without assuming equal responses over space, this approach provides limited inference about causal hypotheses (Williams & Brown, 2019). Assumptions of constant outcomes over time may be problematic for nomadic movement of grassland birds (Green et al., 2019) in response to variable weather patterns (Lipsey & Naugle, 2017). Assuming constant outcomes over time may be a greater issue for CRP effects estimated from a single year (2016) than mean effects of LPCI grazing among 3 years of study. For these reasons, we suggest treatment effects may be best incorporated within adaptive management, where additional monitoring is expected to reduce uncertainty and improve learning (Lyons et al., 2008).

Monitoring effectiveness of conservation on private land to increase populations of imperiled grassland birds is useful for determining management actions that best address recovery objectives (Lyons et al., 2008). By comparing relative population changes to trends used to set population objectives in the PLJV, current investments in CRP and LPCI grazing exceeded or met population objectives for eight priority species over the 30‐year planning cycle (PLJV, 2007) (Appendix S5). For example, estimates of relative population change suggested conservation practices in the study area during 2016 (Figure 3b) contributed 1.0 M (90% CI: 0.6–1.4) Grasshopper Sparrows toward the 5.1 M increase needed to achieve the PLJV (2007) population target by the end of the 30‐year planning cycle. Conservation practices did not meet population objectives for seven species (Appendix S5), and although other practices are needed to reach targets for these species, only Scissor‐tailed Flycatcher showed a negative regional trend (Sauer et al., 2017) (Appendix S1). We suggest treatment effects of practices may be useful in conservation planning for the optimal management of species (Schwartz et al., 2018). As an example of single‐species management, we found a negative effect of LPCI grazing, a positive effect of introduced CRP, and a large positive effect of native CRP on the population density of the Grasshopper Sparrow (Table 1). The 2016 allocation of conservation practices included relatively small extents of LPCI grazing (1887 km^2^) and introduced CRP (1425 km^2^) and large areas of native CRP (13,718 km^2^), resulting in a large net contribution to Grasshopper Sparrow populations (Figure 3).

By addressing threatening processes of habitat loss and degradation in agricultural landscapes (Bowman et al., 2017), conservation on private land provides a solution to one of the most pressing conservation problems for declining avifauna of North America (Rosenberg et al., 2019). Solutions ultimately depend on development of private land conservation programs at the interface between social and natural systems that simultaneously address ecological threats and improve human well‐being (Kareiva & Marvier, 2012). Because wildlife conservation in agricultural production landscapes exists within complex social–ecological systems, a thorough understanding of human dimensions and economic incentives that drive decision‐making processes may be necessary before private land conservation can occur (Knight et al., 2010). In this respect, collaborations between private landowners, government agencies, land managers, and scientists represent a shared vision for coproduction of science in working landscapes (Naugle et al., 2020). Coproduction provides foundations for developing a shared commitment to solve conservation problems, setting priorities for collective stakeholder objectives, evaluating effectiveness of management actions, and delivering on‐the‐ground conservation to achieve the best possible outcomes (Schwartz et al., 2018). Monitoring effectiveness of private land conservation practices increases the confidence of resource professionals and promotes accountability toward meeting intended objectives (Briske et al., 2017). Our results suggested local conservation practices scaled up to meet regional population recovery goals for the majority of imperiled grassland birds of conservation concern. Recovery of grassland bird populations in the Great Plains with majority private land tenure may ultimately depend on social capital to reward private landowners for conserving wildlife in the public trust (Briske et al., 2017).

## Supporting information

Appendix S1. The common name, scientific name, grassland specialization (Vickery & Herkert 1999), breeding‐season vulnerability (Partners in Flight 2019) and trend for the Central Breeding Bird Survey Region (BBS, Sauer et al. 2017) for 35 species encountered in the study area during 2015 – 2017, Colorado, Kansas, New Mexico, Oklahoma and Texas.
Appendix S2. Predictions and references for the hypothesized positive (+), negative (−), equivacal (=), mixed (+/−) and uncertain (?) responses to Native Conservation Reserve Program (CRP) and introduced CRP treatments relative to agricultural reference lands, and Lesser Prairie‐chicken Initiative (LPCI) prescribed grazing relative to reference grasslands for 35 species encountered in the study area, Colorado, Kansas, New Mexico, Oklahoma and Texas, 2015 ‐ 2017
Appendix S3. Point count protocols and distance sampling methods to estimate population density within the study area, Colorado, Kansas, New Mexico, Oklahoma and Texas, 2015 ‐ 2017.
Appendix S4. The population densities ($\hat{D}$ km^‐2^) and Standard Errors (SE) for Lesser Prairie‐chicken Initiative (LPCI)‐prescribed grazing, native Conservation Reserve Program (CRP), introduced CRP, reference grasslands and reference agricultural lands within the study area, Colorado, Kansas, New Mexico, Oklahoma and Texas, 2015 ‐ 2017.
Appendix S5. Mean relative population sizes attributed to treatment effects (${\hat{N}}_{\mathrm{rel}}$), absolute population sizes for species occurring on conservation practices (${\hat{N}}_{\mathrm{abs}}$), mean total population sizes for the study area (${\hat{N}}_{\mathrm{tot}}$), and associated Standard Errors (SE), Colorado, Kansas, New Mexico, Oklahoma and Texas, 2015 ‐ 2017.
Appendix S6. The relative (${\hat{\delta }}_{\mathrm{rel}}$) and absolute (${\hat{\delta }}_{\mathrm{abs}}$) percent contributions to the regional population across conservation practices, Standard Errors (SE), and Lower (LCL) and Upper (UCL) 90% Confidence Limits, respectively in 2016, and the annual trend (%) objective for setting Playa Lakes Joint Venture (PLJV) population targets in the Shortgrass Prairie (BCR 18) and Central Mixed‐grass Prairie (BCR 19) Bird Conservation Regions, Colorado, Kansas, New Mexico, Oklahoma and Texas.
Appendix S7. Model selection for the effects of species vulnerability and grassland obligates on the percentage of populations conserved in the study area, Colorado, Kansas, New Mexico, Oklahoma and Texas, 2016.
