# Supplementary material for: Scaling up private land conservation to meet recovery goals for grassland birds
Source: Conserv Biol. 2021 Jun 30;35(5):1564–74. doi: 10.1111/cobi.13731 (PMC8518544; doi:10.1111/cobi.13731)
Supplement: Supplementary file 1 — Appendix S1. The common name, scientific name, grassland specialization (Vickery & Herkert 1999), breeding‐season vulnerability (Partners in Flight 2019) and trend for the Central Breeding Bird Survey Region (BBS, Sauer et al. 2017) for 35 species encountered in the study area during 2015 – 2017, Colorado, Kansas, New Mexico, Oklahoma and Texas. Appendix S2. Predictions and references for the hypothesized positive (+), negative (−), equivacal (=), mixed (+/−) and uncertain (?) responses to Native Conservation Reserve Program (CRP) and introduced CRP treatments relative to agricultural reference lands, and Lesser Prairie‐chicken Initiative (LPCI) prescribed grazing relative to reference grasslands for 35 species encountered in the study area, Colorado, Kansas, New Mexico, Oklahoma and Texas, 2015 ‐ 2017 Appendix S3. Point count protocols and distance sampling methods to estimate population density within the study area, Colorado, Kansas, New Mexico, Oklahoma and Texas, 2015 ‐ 2017. Appendix S4. The population densities (D^ km‐2) and Standard Errors (SE) for Lesser Prairie‐chicken Initiative (LPCI)‐prescribed grazing, native Conservation Reserve Program (CRP), introduced CRP, reference grasslands and reference agricultural lands within the study area, Colorado, Kansas, New Mexico, Oklahoma and Texas, 2015 ‐ 2017. Appendix S5. Mean relative population sizes attributed to treatment effects (N^rel), absolute population sizes for species occurring on conservation practices (N^abs), mean total population sizes for the study area (N^tot), and associated Standard Errors (SE), Colorado, Kansas, New Mexico, Oklahoma and Texas, 2015 ‐ 2017. Appendix S6. The relative (δ^rel) and absolute (δ^abs) percent contributions to the regional population across conservation practices, Standard Errors (SE), and Lower (LCL) and Upper (UCL) 90% Confidence Limits, respectively in 2016, and the annual trend (%) objective for setting Playa Lakes Joint Venture (PLJV) population targets in the Sho [file COBI-35-1564-s001.docx]

Appendix S1. The common name, scientific name, grassland specialization (Vickery & Herkert 1999), breeding-season vulnerability (Partners in Flight 2019) and trend for the Central Breeding Bird Survey Region (BBS, Sauer et al. 2017) for 35 species encountered in the study area during 2015 – 2017, Colorado, Kansas, New Mexico, Oklahoma and Texas. Breeding-season vulnerability was calculated as the weighted mean of the regional combined scores for Bird Conservation Regions 18, 19 and 35 in the study area. The trend for the Central BBS Region represents the percentage annual change from 1966 to 2015 with 95% Credible Intervals.

| Common name | Scientific name | Specialization | Vulnerability | Trend |
| --- | --- | --- | --- | --- |
| Northern Bobwhite | *Colinus virginianus* | Facultative | 13.79 | -2.61 ( -3.07, -2.23) |
| Scaled Quail | *Callipepla squamata* | Facultative | 15.71 | 0.15 ( -1.22, 1.60) |
| Ring-necked Pheasant | *Phasianus colchicus* | Facultative | 11.93 | -0.03 ( -0.57, 0.46) |
| Mourning Dove | *Zenaida macroura* | Facultative | 11.45 | -0.36 ( -0.53, -0.20) |
| Common Nighthawk | *Chordeiles minor* | Facultative | 12.53 | -0.92 ( -1.36, -0.49) |
| Killdeer | *Charadrius vociferus* | Facultative | 13.12 | -0.28 ( -0.51, -0.06) |
| Long-billed Curlew | *Numenius americanus* | Obligate | 17.07 | -0.90 ( -2.12, 0.08) |
| Turkey Vulture | *Cathartes aura* | Facultative | 8.48 | 1.88 ( 1.37, 2.37) |
| Northern Harrier | *Circus hudsonius* | Obligate | 15.45 | -1.21 ( -1.73, -0.73) |
| Swainson's Hawk | *Buteo swainsoni* | Obligate | 13.86 | 0.43 ( 0.04, 0.81) |
| Burrowing Owl | *Athene cunicularia* | Obligate | 13.45 | -0.86 ( -2.31, 0.19) |
| American Kestrel | *Falco sparverius* | Facultative | 12.41 | -0.60 ( -0.98, -0.22) |
| Ash-throated Flycatcher | *Myiarchus cinerascens* | Facultative | 9.62 | 2.17 ( 1.46, 2.88) |
| Cassin's Kingbird | *Tyrannus vociferans* | Facultative | 11.04 | 4.45 ( 2.08, 6.23) |
| Western Kingbird | *Tyrannus verticalis* | Facultative | 12.29 | 0.20 ( -0.18, 0.55) |
| Eastern Kingbird | *Tyrannus tyrannus* | Facultative | 13.32 | -0.93 ( -1.16, -0.72) |
| Scissor-tailed Flycatcher | *Tyrannus forficatus* | Facultative | 12.60 | -0.78 ( -1.08, -0.47) |
| Say's Phoebe | *Sayornis saya* | Facultative | 11.14 | 1.03 ( 0.46, 1.58) |
| Loggerhead Shrike | *Lanius ludovicianus* | Facultative | 14.45 | -3.41 ( -3.77, -3.06) |
| Chihuahuan Raven | *Corvus cryptoleucus* | Facultative | 13.69 | -0.75 ( -2.12, 0.62) |
| Horned Lark | *Eremophila alpestris* | Obligate | 11.24 | -2.68 ( -3.17, -2.26) |
| Eastern Bluebird | *Sialia sialis* | Facultative | 9.44 | 1.84 ( 1.42, 2.23) |
| Cassin's Sparrow | *Peucaea cassinii* | Obligate | 15.17 | -0.69 ( -2.28, 0.44) |
| Grasshopper Sparrow | *Ammodramus savannarum* | Obligate | 16.43 | -2.02 ( -2.62, -1.48) |
| Lark Sparrow | *Chondestes grammacus* | Facultative | 13.81 | -1.02 ( -1.46, -0.60) |
| Lark Bunting | *Calamospiza melanocorys* | Obligate | 15.56 | -2.87 ( -5.17, -1.31) |
| Field Sparrow | *Spizella pusilla* | Facultative | 11.41 | -1.52 ( -2.00, -1.17) |
| Rufous-crowned Sparrow | *Aimophila ruficeps* | Facultative | 13.07 | -1.67 ( -3.15, -0.15) |
| Eastern Meadowlark | *Sturnella magna* | Obligate | 13.48 | -2.91 ( -3.35, -2.57) |
| Western Meadowlark | *Sturnella neglecta* | Obligate | 13.95 | -1.09 ( -1.29, -0.89) |
| Red-winged Blackbird | *Agelaius phoeniceus* | Facultative | 9.81 | -0.46 ( -0.66, -0.26) |
| Brown-headed Cowbird | *Molothrus ater* | Facultative | 8.81 | -0.12 ( -0.32, 0.09) |
| Brewer's Blackbird | *Euphagus cyanocephalus* | Facultative | 9.00 | -0.66 ( -1.17, -0.20) |
| Common Yellowthroat | *Geothlypis trichas* | Facultative | 8.60 | -1.12 ( -1.34, -0.90) |
| Dickcissel | *Spiza americana* | Obligate | 12.26 | -0.19 ( -0.73, 0.25) |

Appendix S2. Predictions and references for the hypothesized positive (+), negative (−), equivacal (=), mixed (+/−) and uncertain (?) responses to Native Conservation Reserve Program (CRP) and introduced CRP treatments relative to agricultural reference lands, and Lesser Prairie-chicken Initiative (LPCI) prescribed grazing relative to reference grassands for 35 species encountered in the study area, Colorado, Kansas, New Mexico, Oklahoma and Texas, 2015 - 2017.

| Common name | Native CRP | Introduced CRP | LPCI grazing | Reference |
| --- | --- | --- | --- | --- |
| Northern Bobwhite | **+** | **−** | **+** | (Sands et al. 2012; Brennan et al. 2020) |
| Scaled Quail | **+** | **−** | **+** | (Fulbright et al. 2019; Dabbert et al. 2020) |
| Ring-necked Pheasant | **−** | **−** | **+** | (Taylor et al. 2018; Giudice & Ratti 2020) |
| Mourning Dove | **++** | **+** | **+** | (Riffell et al. 2008b; Otis et al. 2020) |
| Common Nighthawk | **+** | **+** | **−** | (Riffell et al. 2008b; Brigham et al. 2020) |
| Killdeer | **−** | **−** | **−** | (Jackson & Jackson 2020) |
| Long-billed Curlew | **++** | **+** | **−** | (Bakker & Higgins 2009; Fellows & Jones 2009; Dugger & Dugger 2020) |
| Turkey Vulture | **+** | **+** | **+** | (Kirk & Mossman 2020) |
| Northern Harrier | **++** | **+** | **+** | (Riffell et al. 2008b; Smith et al. 2020) |
| Swainson's Hawk | **++** | **+** | **+** | (Riffell et al. 2008b; Wiggins et al. 2014) |
| Burrowing Owl | **=** | **=** | **−** | (Poulin et al. 2020) |
| American Kestrel | **+** | **+** | **+** | (Bock et al. 1993; Riffell et al. 2008b) |
| Ash-throated Flycatcher | **=** | **=** | **+** | (Cardiff & Dittmann 2020) |
| Cassin's Kingbird | **?** | **?** | **?** | (Tweit & Tweit 2020) |
| Western Kingbird | **=** | **=** | **+** | (USDA 2006; Riffell et al. 2008b) |
| Eastern Kingbird | **+** | **+** | **+** | (USDA 2006; Riffell et al. 2008a) |
| Scissor-tailed Flycatcher | **+** | **?** | **+** | (Klute et al. 1997; Chapman et al. 2004) |
| Say's Phoebe | **?** | **?** | **=** | (Schukman & Wolf 2020) |
| Loggerhead Shrike | **=** | **=** | **+** | (Riffell et al. 2008a; Yosef 2020) |
| Chihuahuan Raven | **−** | **−** | **−** | (Wiggins 2018) |
| Horned Lark | **−** | **−−** | **−** | (Bakker & Higgins 2009; Beason 2020) |
| Eastern Bluebird | **=** | **=** | **−** | (Riffell et al. 2008a; Gowaty & Plissner 2020) |
| Cassin's Sparrow | **++** | **+** | **+** | (Bock et al. 1993; Bakker & Higgins 2009; Thompson et al. 2009) |
| Grasshopper Sparrow | **++** | **+** | **+/−** | (Bock et al. 1993; Bakker & Higgins 2009; Herkert 2009) |
| Lark Sparrow | **+** | **+** | **−** | (Bock et al. 1993; Herkert 2009) |
| Lark Bunting | **++** | **+** | **+/−** | (Bock et al. 1993; Johnson & Schwartz 1993; Bakker & Higgins 2009) |
| Field Sparrow | **+** | **+** | **+** | (Walk & Warner 2000; Carey et al. 2020) |
| Rufous-crowned Sparrow | **?** | **?** | **+** | (Collins 2020) |
| Eastern Meadowlark | **++** | **+** | **+** | (Riffell et al. 2008b; Bakker & Higgins 2009; Jaster et al. 2020) |
| Western Meadowlark | **++** | **+** | **+/−** | (Bock et al. 1993; Riffell et al. 2008b; Bakker & Higgins 2009) |
| Red-winged Blackbird | **−** | **−** | **+/−** | (Bock et al. 1993; Yasukawa & Searcy 2020) |
| Brown-headed Cowbird | **−** | **−** | **=** | (Bock et al. 1993; Lowther 2020) |
| Brewer's Blackbird | **=** | **=** | **−** | (Bock et al. 1993; Riffell et al. 2008b) |
| Common Yellowthroat | **=** | **=** | **+** | (Bock et al. 1993; Riffell et al. 2008b) |
| Dickcissel | **=** | **=** | **+/−** | (Bock et al. 1993; Riffell et al. 2008b) |

Appendix S3. Point count protocols and distance sampling methods to estimate population density within the study area, Colorado, Kansas, New Mexico, Oklahoma and Texas, 2015 - 2017.

The sampling protocols for avian monitoring involved a two-stage design with systematic sub-samples of 16 nested within 1 km^2^ grid cells, with point count plots located 250 m apart and ≥125 m from the grid cell boundaries (Pavlacky et al. 2017). We monitored breeding season abundance of adult bird species using 6-min point counts between 20 April and 15 June, and from one-half hour before sunrise to five hours after sunrise at each accessible point count location (Pavlacky et al. 2017). Field technicians measured distances to each bird detection using a laser rangefinder (Pavlacky et al. 2012).

We developed *a priori* hypotheses for conservation practices designed to address threatening processes, synthesized casual predictions for population responses to conservation practices based on habitat requirements of species (Appendix S2), compared predictions to observed treatment effects (Table 1), and used inductive reasoning to make inferences about the hypotheses (Williams 1997; Ford & Ishii 2001).

We estimated population density seperately for each species, treatment and reference strata, year and study area using a sequential framework where 1) year-specific detection functions were applied to species with ≥ 80 detections per year, 2) global detection functions were applied to species with < 80 detections per year and ≥ 80 detections over the years of the project, and 3) remedial measures were used for species with moderate departures from the assumptions of distance sampling (Buckland et al. 2001; Pavlacky et al. 2017). We fit continuous models with no series expansions to all species and using the recommended 10% truncation for point transects (Buckland et al. 2001). For the year-specific detection functions, we fit Conventional Distance Sampling models using the half-normal and hazard-rate key functions with no series expansions (Thomas et al. 2010). For the global detection functions, in addition to the above models, we fit Multiple Covariate Distance Sampling models using half-normal and hazard-rate key function models with a factor year covariate and no series expansions (Thomas et al. 2010). We selected the best fitting detection function for each species using Akaike’s Information Criterion adjusted for sample size (AIC*_c_*, Burnham and Anderson 2002, Thomas et al. 2010) and considered the most parsimonious model as the estimation model.

Appendix S4. The population densities ($\hat{\text{D}}$ km^-2^) and Standard Errors (SE) for Lesser Prairie-chicken Initiative (LPCI)-prescribed grazing, native Conservation Reserve Program (CRP), introduced CRP, reference grasslands and reference agricultural lands within the study area, Colorado, Kansas, New Mexico, Oklahoma and Texas, 2015 - 2017. Population densities for native and introduced CRP were estimated for 2016.

| Species | LPCI | | Native CRP | | Intro. CRP | | Grassland | | Ag. land | |
| --- | --- | --- | --- | --- | --- | --- | --- | --- | --- | --- |
|  | $\hat{\text{D}}$ | SE | $\hat{\text{D}}$ | SE | $\hat{\text{D}}$ | SE | $\hat{\text{D}}$ | SE | $\hat{\text{D}}$ | SE |
| Northern Bobwhite | 5.05 | 0.60 | 3.60 | 1.13 | 9.06 | 1.43 | 3.00 | 0.41 | 3.70 | 0.60 |
| Scaled Quail | 0.79 | 0.23 | 1.30 | 0.65 | 2.17 | 0.75 | 1.97 | 0.68 | 0.29 | 0.15 |
| Ring-necked Pheasant | 0.36 | 0.11 | 1.09 | 0.27 | 0.35 | 0.16 | 0.33 | 0.07 | 3.80 | 0.37 |
| Mourning Dove | 10.25 | 0.73 | 13.02 | 1.56 | 20.22 | 1.97 | 7.36 | 0.61 | 10.12 | 0.74 |
| Common Nighthawk | 1.53 | 0.26 | 0.11 | 0.05 | 0.37 | 0.21 | 0.78 | 0.18 | 1.60 | 0.76 |
| Killdeer | 1.40 | 0.37 | 0.53 | 0.30 | 0.80 | 0.26 | 1.37 | 0.28 | 6.09 | 0.88 |
| Long-billed Curlew | 0.06 | 0.06 | 0.04 | 0.04 | - | - | 0.17 | 0.15 | 0.06 | 0.05 |
| Turkey Vulture | 1.67 | 0.72 | - | - | 0.05 | 0.05 | 0.26 | 0.08 | 0.19 | 0.15 |
| Northern Harrier | 0.03 | 0.03 | 0.22 | 0.14 | 0.07 | 0.07 | - | - | - | - |
| Swainson's Hawk | 0.09 | 0.03 | 0.12 | 0.07 | 0.15 | 0.08 | 0.04 | 0.01 | 0.20 | 0.08 |
| Burrowing Owl | 0.08 | 0.04 | 0.02 | 0.03 | - | - | 0.19 | 0.20 | - | - |
| American Kestrel | 0.15 | 0.07 | 0.04 | 0.04 | 0.44 | 0.22 | 0.13 | 0.05 | 0.06 | 0.03 |
| Ash-throated Flycatcher | 0.04 | 0.04 | - | - | 0.80 | 0.62 | 0.18 | 0.13 | - | - |
| Cassin's Kingbird | 0.04 | 0.05 | - | - | - | - | 0.05 | 0.05 | - | - |
| Western Kingbird | 3.52 | 0.53 | 3.07 | 1.14 | 12.08 | 2.59 | 4.94 | 1.07 | 5.58 | 1.50 |
| Eastern Kingbird | 3.36 | 1.04 | 0.25 | 0.27 | - | - | 0.24 | 0.15 | - | - |
| Scissor-tailed Flycatcher | 1.16 | 0.44 | 0.99 | 0.72 | 1.94 | 0.62 | 1.91 | 0.47 | 1.24 | 0.31 |
| Say's Phoebe | 0.03 | 0.03 | 0.11 | 0.06 | 0.03 | 0.03 | 0.03 | 0.03 | - | - |
| Loggerhead Shrike | 0.12 | 0.06 | 0.07 | 0.07 | 0.20 | 0.14 | 0.07 | 0.04 | - | - |
| Chihuahuan Raven | 0.19 | 0.07 | 0.20 | 0.11 | 0.32 | 0.18 | 0.31 | 0.14 | 0.09 | 0.04 |
| Horned Lark | 19.11 | 2.29 | 47.65 | 10.77 | 23.10 | 8.79 | 47.75 | 5.78 | 78.02 | 7.44 |
| Eastern Bluebird | 0.33 | 0.14 | - | - | 0.27 | 0.16 | - | - | - | - |
| Cassin's Sparrow | 47.05 | 1.83 | 26.35 | 4.45 | 47.74 | 5.12 | 33.67 | 4.92 | 2.55 | 0.46 |
| Grasshopper Sparrow | 44.83 | 5.22 | 104.62 | 13.82 | 71.97 | 13.64 | 64.38 | 5.80 | 33.63 | 6.65 |
| Lark Sparrow | 16.48 | 1.88 | 2.72 | 1.38 | 6.01 | 2.07 | 18.25 | 3.56 | 2.70 | 0.76 |
| Lark Bunting | 9.32 | 1.14 | 25.23 | 9.93 | 3.85 | 1.82 | 14.76 | 3.43 | 12.36 | 5.66 |
| Field Sparrow | 1.32 | 0.39 | 0.07 | 0.05 | 1.07 | 0.65 | 0.14 | 0.08 | - | - |
| Rufous-crowned Sparrow | 0.34 | 0.23 | - | - | - | - | - | - | - | - |
| Eastern Meadowlark | 18.63 | 1.70 | 13.06 | 4.04 | 25.09 | 3.40 | 6.85 | 1.30 | 4.64 | 1.08 |
| Western Meadowlark | 12.31 | 1.03 | 19.92 | 2.69 | 13.32 | 3.20 | 22.29 | 1.73 | 21.43 | 2.04 |
| Red-winged Blackbird | 5.36 | 0.68 | 1.41 | 0.60 | 4.97 | 2.19 | 5.19 | 1.41 | 32.61 | 5.43 |
| Brown-headed Cowbird | 8.85 | 1.09 | 1.51 | 0.84 | 3.93 | 1.25 | 8.15 | 1.58 | 25.71 | 4.12 |
| Brewer's Blackbird | 0.06 | 0.06 | - | - | 0.17 | 0.17 | - | - | - | - |
| Common Yellowthroat | 0.12 | 0.07 | - | - | - | - | - | - | - | - |
| Dickcissel | 11.30 | 2.75 | 3.08 | 1.52 | 1.54 | 0.91 | 8.78 | 1.92 | 26.45 | 5.40 |

Appendix S5. Mean relative population sizes attributed to treatment effects ($\hat{N}_{\text{rel}}$), absolute population sizes for species occurring on conservation practices ($\hat{N}_{\text{abs}}$), mean total population sizes for the study area ($\hat{N}_{\text{tot}}$), and associated Standard Errors (SE), Colorado, Kansas, New Mexico, Oklahoma and Texas, 2015 - 2017.

| Species | Relative | | Absolute | | Study area | |
| --- | --- | --- | --- | --- | --- | --- |
|  | ${\hat{\text{N}}}_{\text{rel}}$ | SE | ${\hat{\text{N}}}_{\text{abs}}$ | SE | ${\hat{\text{N}}}_{\text{tot}}$ | SE |
| Northern Bobwhite | 10,106 | 17,799 | 71,831 | 15,722 | 756,698 | 70,006 |
| Scaled Quail | 14,194 | 9,342 | 22,370 | 9,020 | 318,313 | 59,754 |
| Ring-necked Pheasant | -42,026 | 6,347 | 16,191 | 3,694 | 351,424 | 36,440 |
| Mourning Dove | 59,560 | 23,931 | 226,699 | 21,628 | 1,770,623 | 111,105 |
| Common Nighthawk | -20,786 | 10,480 | 4,889 | 905 | 120,768 | 45,917 |
| Killdeer | -83,876 | 12,778 | 10,997 | 4,156 | 546,451 | 77,297 |
| Long-billed Curlew | -652 | 894 | 593 | 530 | 12,560 | 6,984 |
| Turkey Vulture | -126 | 2,447 | 3,208 | 1,362 | 36,769 | 10,613 |
| Northern Harrier | - | - | 3,193 | 1,982 | 10,376 | 6,828 |
| Swainson's Hawk | -950 | 1,484 | 2,096 | 976 | 18,005 | 4,840 |
| Burrowing Owl | -203 | 386 | 498 | 354 | 18,767 | 7,078 |
| American Kestrel | 244 | 709 | 1,405 | 608 | 15,548 | 4,318 |
| Ash-throated Flycatcher | 871 | 915 | 1,221 | 882 | 107,452 | 30,670 |
| Cassin’s Kingbird | -11 | 123 | 81 | 85 | 23,266 | 11,450 |
| Western Kingbird | -27,788 | 26,301 | 65,975 | 16,135 | 1,161,421 | 160,578 |
| Eastern Kingbird | 5,873 | 1,976 | 9,811 | 4,195 | 539,851 | 230,463 |
| Scissor-tailed Flycatcher | -3,827 | 10,847 | 18,501 | 9,951 | 481,604 | 86,518 |
| Say's Phoebe | 4 | 81 | 1,605 | 852 | 23,705 | 11,453 |
| Loggerhead Shrike | 1,372 | 1,054 | 1,519 | 1,051 | 24,146 | 7,911 |
| Chihuahuan Raven | 1,571 | 1,718 | 3,498 | 1,589 | 29,809 | 10,755 |
| Horned Lark | -548,846 | 180,714 | 722,643 | 148,374 | 7,679,778 | 781,981 |
| Eastern Bluebird | - | - | 1,005 | 356 | 134,044 | 36,753 |
| Cassin's Sparrow | 416,121 | 62,567 | 518,217 | 61,553 | 2,701,367 | 314,686 |
| Grasshopper Sparrow | 991,547 | 211,968 | 1,622,293 | 190,784 | 7,392,968 | 820,292 |
| Lark Sparrow | 1,569 | 23,102 | 76,947 | 19,490 | 916,608 | 137,023 |
| Lark Bunting | 154,164 | 157,235 | 369,149 | 136,326 | 1,580,541 | 317,385 |
| Field Sparrow | 2,231 | 745 | 4,962 | 1,371 | 37,491 | 12,387 |
| Rufous-crowned Sparrow | - | - | 643 | 431 | 36,287 | 12,940 |
| Eastern Meadowlark | 166,896 | 57,738 | 250,062 | 55,729 | 970,943 | 153,368 |
| Western Meadowlark | -51,024 | 46,693 | 315,516 | 37,170 | 3,352,900 | 262,372 |
| Red-winged Blackbird | -466,993 | 75,474 | 36,548 | 8,884 | 3,673,889 | 661,499 |
| Brown-headed Cowbird | -361,676 | 58,102 | 43,040 | 11,884 | 3,009,038 | 395,090 |
| Brewer’s Blackbird | - | - | 347 | 265 | 7,302 | 3,309 |
| Common Yellowthroat | - | - | 231 | 135 | 6,726 | 4,833 |
| Dickcissel | -351,349 | 77,548 | 65,793 | 21,472 | 3,093,410 | 503,202 |

Appendix S6. The relative (${\hat{\text{δ}}}_{\text{rel}}$) and absolute (${\hat{\text{δ}}}_{\text{abs}}$) percent contributions to the regional population across conservation practices, Standard Errors (SE), and Lower (LCL) and Upper (UCL) 90% Confidence Limits, respectively in 2016, and the annual trend (%) objective for setting Playa Lakes Joint Venture (PLJV) population targets in the Shortgrass Prairie (BCR 18) and Central Mixed-grass Prairie (BCR 19) Bird Conservation Regions, Colorado, Kansas, New Mexico, Oklahoma and Texas. In 2016, 10.53% of the land area was enrolled the conservation practices.

| Species | Relative contribution | | | | Absolute contribution | | | | PLJV | |
| --- | --- | --- | --- | --- | --- | --- | --- | --- | --- | --- |
|  | ${\hat{\text{δ}}}_{\text{rel}}$ | SE | LCL | UCL | ${\hat{\text{δ}}}_{\text{abs}}$ | SE | LCL | UCL | 18 | 19 |
| Northern Bobwhite | 1.34 | 2.36 | 0.28 | 7.89 | 9.49 | 2.26 | 6.11 | 13.52 | 0.1 | 0.1 |
| Scaled Quail | 4.46 | 3.05 | 0.82 | 10.78 | 7.03 | 3.13 | 2.78 | 13.01 | 0.1 | 0.1 |
| Ring-necked Pheasant | -11.96 | 2.19 | -15.80 | -8.59 | 4.61 | 1.15 | 2.89 | 6.70 | -1.8 | 0.1 |
| Mourning Dove | 3.36 | 1.37 | 1.48 | 5.98 | 12.80 | 1.46 | 10.49 | 15.31 |  |  |
| Common Nighthawk | -17.21 | 10.87 | -38.04 | -3.60 | 4.05 | 1.71 | 1.71 | 7.33 |  |  |
| Killdeer | -15.35 | 3.19 | -20.95 | -10.48 | 2.01 | 0.81 | 0.89 | 3.57 |  |  |
| Long-billed Curlew | -5.19 | 7.68 | -24.23 | -0.30 | 4.72 | 4.97 | 0.06 | 16.03 | -2.3 | - |
| Turkey Vulture | -0.34 | 6.66 | -70.27 | 0.00 | 8.72 | 4.48 | 2.83 | 17.42 |  |  |
| Northern Harrier | - | - | - | - | 30.77 | 27.84 | 0.84 | 78.13 |  |  |
| Swainson's Hawk | -5.28 | 8.37 | -26.40 | -0.57 | 11.64 | 6.26 | 3.48 | 23.73 | 0.1 | -2.3 |
| Burrowing Owl | -1.08 | 2.09 | -7.16 | -0.39 | 2.65 | 2.14 | 0.29 | 7.27 |  |  |
| American Kestrel | 1.57 | 4.58 | 0.01 | 17.29 | 9.04 | 4.64 | 2.93 | 18.04 |  |  |
| Ash-throated Flycatcher | 0.81 | 0.88 | 0.00 | 2.90 | 1.14 | 0.88 | 0.14 | 3.05 |  |  |
| Cassin’s Kingbird | -0.05 | 0.53 | -4.79 | 0.00 | 0.35 | 0.41 | 0.00 | 1.34 |  |  |
| Western Kingbird | -2.39 | 2.29 | -7.56 | -0.10 | 5.68 | 1.60 | 3.34 | 8.59 |  |  |
| Eastern Kingbird | 1.09 | 0.59 | 0.33 | 2.28 | 1.82 | 1.10 | 0.45 | 4.06 |  |  |
| Scissor-tailed Flycatcher | -0.79 | 2.26 | -1.43 | -8.63 | 3.84 | 2.18 | 1.07 | 8.21 | 1.7 | -2.3 |
| Say's Phoebe | 0.02 | 0.34 | 0.00 | 5.52 | 6.77 | 4.86 | 1.08 | 16.81 |  |  |
| Loggerhead Shrike | 5.68 | 4.75 | 0.51 | 15.85 | 6.29 | 4.82 | 0.81 | 16.39 | 0.1 | -2.3 |
| Chihuahuan Raven | 5.27 | 6.07 | 0.00 | 19.32 | 11.73 | 6.81 | 3.05 | 25.01 |  |  |
| Horned Lark | -7.15 | 2.46 | -11.72 | -3.64 | 9.41 | 2.16 | 6.17 | 13.25 |  |  |
| Eastern Bluebird | - | - | - | - | 0.75 | 0.34 | 0.29 | 1.41 |  |  |
| Cassin's Sparrow | 15.40 | 2.93 | 10.90 | 20.52 | 19.18 | 3.19 | 14.22 | 24.70 | -0.9 | -2.3 |
| Grasshopper Sparrow | 13.41 | 3.23 | 8.56 | 19.15 | 21.94 | 3.55 | 16.40 | 28.04 | -2.3 | -1.4 |
| Lark Sparrow | 0.17 | 2.52 | 0.00 | 26.69 | 8.39 | 2.47 | 4.79 | 12.89 | 0.1 | -2.3 |
| Lark Bunting | 9.75 | 10.14 | 0.13 | 31.77 | 23.36 | 9.82 | 9.51 | 41.04 | -2.3 | -2.3 |
| Field Sparrow | 5.95 | 2.80 | 2.21 | 11.35 | 13.24 | 5.70 | 5.37 | 23.90 |  |  |
| Rufous-crowned Sparrow | - | - | - | - | 1.77 | 1.35 | 0.24 | 4.66 |  |  |
| Eastern Meadowlark | 17.19 | 6.54 | 7.90 | 29.13 | 25.75 | 7.04 | 15.16 | 38.04 | 0.1 | -1.1 |
| Western Meadowlark | 1.52 | 1.40 | 0.08 | 4.67 | 9.41 | 1.33 | 7.33 | 11.72 |  |  |
| Red-winged Blackbird | -12.71 | 3.08 | -18.19 | -8.10 | 0.99 | 0.30 | 0.56 | 1.56 |  |  |
| Brown-headed Cowbird | -12.02 | 2.49 | -16.42 | -8.23 | 1.43 | 0.44 | 0.80 | 2.24 |  |  |
| Brewer’s Blackbird | - | - | - | - | 4.75 | 4.21 | 0.32 | 13.94 |  |  |
| Common Yellowthroat | - | - | - | - | 3.44 | 3.19 | 0.18 | 10.52 |  |  |
| Dickcissel | -11.36 | 3.11 | -16.97 | -6.76 | 2.13 | 0.78 | 1.04 | 3.59 | 3.5 | 0.1 |

Appendix S7. Model selection for the effects of species vulnerability and grassland obligates on the percentage of populations conserved in the study area, Colorado, Kansas, New Mexico, Oklahoma and Texas, 2016.

| Model | log(*L*) | *K* | AIC*_c_* | ΔAIC*_c_* | *w_i_* |
| --- | --- | --- | --- | --- | --- |
| log*_e_*(Vulnerability) + Obligate | -123.06 | 4 | 255.33 | 0.00 | 0.529 |
| log*_e_*(Vulnerability) * Obligate | -122.45 | 5 | 256.78 | 1.45 | 0.256 |
| Obligate | -125.56 | 3 | 257.82 | 2.49 | 0.152 |
| log*_e_*(Vulnerability) | -126.46 | 3 | 259.62 | 4.29 | 0.062 |

**Literature Cited**

Bakker KK, Higgins KF. 2009. Planted grasslands and native sod prairie: equivalent habitat for grassland birds? Western North American Naturalist **69**:235-242.

Beason RC. 2020. Horned Lark (*Eremophila alpestris*), version 1.0 in Billerman SM, editor. Birds of the World. Cornell Lab of Ornithology, Ithaca, New York, USA. Available from <https://doi-org.ezproxy2.library.colostate.edu/10.2173/bow.horlar.01> (accessed July 2020).

Bock CE, Saab VA, Rich TD, Dobkin DS. 1993. Effects of livestock grazing on neotropical migratory landbirds in western North America in Finch DM, and Stangel PW, editors. Status and management of neotropical migratory birds. Technical Report RM-229. United States Department of Agriculture, Forest Service, Rocky Mountain Forest and Range Experiment Station, Fort Collins, Colorado, USA. Available from <https://www.fs.usda.gov/treesearch/pubs/22913> (accessed July 2020).

Brennan LA, Hernandez F, Williford D. 2020. Northern Bobwhite (*Colinus virginianus*), version 1.0 in Poole AF, editor. Birds of the World. Cornell Laboratory of Ornithology, Ithaca, New York, USA. Available from <https://doi-org.ezproxy2.library.colostate.edu/10.2173/bow.norbob.01> (accessed July 2020).

Brigham RM, Ng J, Poulin RG, Grindal SD. 2020. Common Nighthawk (*Chordeiles minor*), version 1.0 in Poole AF, editor. Birds of the World Cornell Lab of Ornithology, Ithaca, New York, USA. Available from <https://doi-org.ezproxy2.library.colostate.edu/10.2173/bow.comnig.01> (accessed July 2020).

Buckland ST, Anderson DR, Burnham KP, Laake JL, Borchers DL, Thomas L 2001. Introduction to distance sampling: estimating abundance of biological populations. Oxford University Press, Oxford, UK.

Burnham KP, Anderson DR 2002. Model selection and multimodel inference: a practical information-theoretic approach. Springer-Verlag, New York, New York, USA.

Cardiff SW, Dittmann DL. 2020. Ash-throated Flycatcher (*Myiarchus cinerascens*), version 1.0 in Poole AF, and Gill FB, editors. Birds of the World Cornell Lab of Ornithology, Ithaca, New York, USA. Available from <https://doi-org.ezproxy2.library.colostate.edu/10.2173/bow.astfly.01> (accessed July 2020).

Carey M, Burhans DE, Nelson DA. 2020. Field Sparrow (*Spizella pusilla*), version 1.0 in Poole AF, editor. Birds of the World. Cornell Laboratory of Ornithology, Ithaca, New York, USA. Available from <https://doi-org.ezproxy2.library.colostate.edu/10.2173/bow.fiespa.01> (accessed July 2020).

Chapman RN, Engle DM, Masters RE, Leslie DM. 2004. Grassland vegetation and bird communities in the southern Great Plains of North America. Agriculture Ecosystems & Environment **104**:577-585.

Collins PW. 2020. Rufous-crowned Sparrow (*Aimophila ruficeps*), version 1.0 in Poole AF, and Gill FB, editors. Birds of the World. Cornell Laboratory of Ornithology, Ithaca, New York, USA. Available from <https://doi-org.ezproxy2.library.colostate.edu/10.2173/bow.rucspa.01> (accessed July 2020).

Dabbert CB, Pleasant G, Schemnitz SD. 2020. Scaled Quail (*Callipepla squamata*), version 1.0 in Poole AF, editor. Birds of the World Cornell Lab of Ornithology, Ithaca, New York, USA. Available from <https://doi-org.ezproxy2.library.colostate.edu/10.2173/bow.scaqua.01> (accessed July 2020).

Dugger BD, Dugger KM. 2020. Long-billed Curlew (*Numenius americanus*), version 1.0 in Poole AF, and Gill FB, editors. Birds of the World Cornell Lab of Ornithology, Ithaca, New York, USA. Available from <https://doi-org.ezproxy2.library.colostate.edu/10.2173/bow.lobcur.01> (accessed July 2020).

Fellows SD, Jones SL 2009. Status assessment and conservation action plan for the long-billed curlew (*Numenius americanus*). Biological Technical Publication, FWS/BTP-R6012-2009. United States Department of Interior, Fish and Wildlife Service, Washington, D.C., USA. Available from <https://www.fws.gov/migratorybirds/pdf/management/focal-species/Long-billedCurlew.pdf> (accessed July 2020).

Ford ED, Ishii H. 2001. The method of synthesis in ecology. Oikos **93**:153-160.

Fulbright TE, Kline HN, Wester DB, Grahmann ED, Hernandez F, Brennan LA, Hehman MW. 2019. Non-native grasses reduce scaled quail habitat. Journal of Wildlife Management **83**:1581-1591.

Giudice JH, Ratti JT. 2020. Ring-necked Pheasant (*Phasianus colchicus*), version 1.0 in Billerman SM, editor. Birds of the World. Cornell Lab of Ornithology, Ithaca, New York, USA. Available from <https://doi-org.ezproxy2.library.colostate.edu/10.2173/bow.rinphe.01> (accessed July 2020).

Gowaty PA, Plissner JH. 2020. Eastern Bluebird (*Sialia sialis*), version 1.0 in Poole AF, editor. Birds of the World. Cornell Lab of Ornithology, Ithaca, New York, USA. Available from <https://doi-org.ezproxy2.library.colostate.edu/10.2173/bow.easblu.01> (accessed July 2020).

Herkert JR. 2009. Response of bird populations to farmland set-aside programs. Conservation Biology **23**:1036-1040.

Jackson BJ, Jackson JA. 2020. Killdeer (*Charadrius vociferus*), version 1.0 in Poole AF, and Gill FB, editors. Birds of the World. Ithaca, New York, USA. Available from <https://doi-org.ezproxy2.library.colostate.edu/10.2173/bow.killde.01> (accessed July 2020), Cornell Lab of Ornithology.

Jaster LA, Jensen WE, Lanyon WE. 2020. Eastern Meadowlark (*Sturnella magna*), version 1.0 in Poole AF, editor. Birds of the World. Cornell Laboratory of Ornithology, Ithaca, New York, USA. Available from <https://doi-org.ezproxy2.library.colostate.edu/10.2173/bow.easmea.01> (accessed July 2020).

Johnson DH, Schwartz MD. 1993. The Conservation Reserve Program: habitat for grassland birds. Great Plains Research **3**:273-295.

Kirk DA, Mossman MJ. 2020. Turkey Vulture (*Cathartes aura*), version 1.0 in Poole AF, and Gill FB, editors. Birds of the World Cornell Lab of Ornithology, Ithaca, New York, USA. Available from <https://doi-org.ezproxy2.library.colostate.edu/10.2173/bow.turvul.01> (accessed July 2020).

Klute DS, Robel RJ, Kemp KE. 1997. Will conversion of Conservation Reserve Program (CRP) lands to pasture be detrimental for grassland birds in Kansas? The American Midland Naturalist **137**:206-212.

Lowther PE. 2020. Brown-headed Cowbird (*Molothrus ater*), version 1.0 in Poole AF, and Gill FB, editors. Birds of the World. Ithaca, New York, USA. Available from <https://doi-org.ezproxy2.library.colostate.edu/10.2173/bow.bnhcow.01> (accessed July 2020), Cornell Lab of Ornithology.

Otis DL, Schulz JH, Miller D, Mirarchi RE, Baskett TS. 2020. Mourning Dove (*Zenaida macroura*), version 1.0 in Poole AF, editor. Birds of the World Cornell Lab of Ornithology, Ithaca, New York, USA. Available from <https://doi-org.ezproxy2.library.colostate.edu/10.2173/bow.moudov.01> (accessed July 2020).

Partners in Flight 2019. Avian Conservation Assessment Database, version 2019. Available from <http://pif.birdconservancy.org/acad> (accessed November 2019).

Pavlacky DC, Jr., Blakesley JA, White GC, Hanni DJ, Lukacs PM. 2012. Hierarchical multi-scale occupancy estimation for monitoring wildlife populations. Journal of Wildlife Management **76**:154–162.

Pavlacky DC, Jr., Lukacs PM, Blakesley JA, Skorkowsky RC, Klute DS, Hahn BA, Dreitz VJ, George TL, Hanni DJ. 2017. A statistically rigorous sampling design to integrate avian monitoring and management within Bird Conservation Regions. PLOS ONE **12**:e0185924.

Poulin RG, Todd LD, Haug EA, Millsap BA, Martell MS. 2020. Burrowing Owl (*Athene cunicularia*), version 1.0 in Poole AF, editor. Birds of the World Cornell Lab of Ornithology, Ithaca, New York, USA. Available from <https://doi-org.ezproxy2.library.colostate.edu/10.2173/bow.burowl.01> (accessed July 2020).

Riffell S, Scognamillo D, Burger LW. 2008a. Effects of the Conservation Reserve Program on northern bobwhite and grassland birds. Environmental Monitoring and Assessment **146**:309-323.

Riffell SK, McIntyre NE, Hayes R. 2008b. Agricultural set-aside programs and grassland birds: insights from broad-scale population trends. Landscape Online **8**:1-20.

Sands JP, Brennan LA, Hernandez F, Kuvlesky WP, Gallagher JF, Ruthven DC. 2012. Impacts of introduced grasses on breeding season habitat use by northern bobwhite in the South Texas plains. Journal of Wildlife Management **76**:608-618.

Sauer JR, Niven DK, Hines JE, D. J. Ziolkowski Jr., Pardieck KL, Fallon JE, Link WA 2017. The North American Breeding Bird Survey, results and analysis 1966 - 2015. Version 2.07.2017. United States Geological Survey, Patuxent Wildlife Research Center, Laurel, Maryland, USA. Available from <https://www.mbr-pwrc.usgs.gov/bbs> (accessed December 2019)

Schukman JM, Wolf BO. 2020. Say's Phoebe (*Sayornis saya*), version 1.0 in Rodewald PG, editor. Birds of the World Cornell Lab of Ornithology, Ithaca, New York, USA. Available from <https://doi-org.ezproxy2.library.colostate.edu/10.2173/bow.saypho.01> (accessed July 2020).

Smith KG, Wittenberg SR, Macwhirter RB, Bildstein KL. 2020. Northern Harrier (*Circus hudsonius*), version 1.0 in Poole AF, and Gill FB, editors. Birds of the World Cornell Lab of Ornithology, Ithaca, New York, USA. Available from <https://doi-org.ezproxy2.library.colostate.edu/10.2173/bow.norhar2.01> (accessed July 2020).

Taylor JS, Bogenschutz TR, Clark WR. 2018. Pheasant responses to US cropland conversion programs: a review and recommendations. Wildlife Society Bulletin **42**:184-194.

Thomas L, Buckland ST, Rexstad EA, Laake JL, Strindberg S, Hedley SL, Bishop JRB, Marques TA, Burnham KP. 2010. Distance software: design and analysis of distance sampling surveys for estimating population size. Journal of Applied Ecology **47**:5-14.

Thompson TR, Boal CW, Lucia D. 2009. Grassland bird associations with introduced and native grass conservation reserve program fields in the southern high plains. Western North American Naturalist **69**:481-490.

Tweit RC, Tweit JC. 2020. Cassin's Kingbird (*Tyrannus vociferans*), version 1.0 in Poole AF, and Gill FB, editors. Birds of the World Cornell Lab of Ornithology, Ithaca, New York, USA. Available from <https://doi-org.ezproxy2.library.colostate.edu/10.2173/bow.caskin.01> (accessed July 2020).

United States Department of Agriculture (USDA) 2006. Migratory bird responses to grazing: Wetlands Reserve Program Grassland Workgroup Report Natural Resources Conservation Service, Washington, D.C., USA. Available from <https://directives.sc.egov.usda.gov/OpenNonWebContent.aspx?content=18514.wba> (accessed July 2020).

Vickery PD, Herkert JR 1999. Ecology and conservation of grassland birds of the Western Hemisphere. Studies in Avian Biology (no. 19). Cooper Ornithological Society, Lawrence, Kansas, USA.

Walk JW, Warner RE. 2000. Grassland management for the conservation of songbirds in the Midwestern USA. Biological Conservation **94**:165-172.

Wiggins DA. 2018. Differential breeding range shifts of ravens (*Corvus* spp.) on the southern Great Plains. Pages 75-84 in Shuford WD, Gill Jr RE, and Handel CM, editors. Trends and traditions: Avifaunal change in western North America. Studies of Western Birds 3. Western Field Ornithologists, Camarillo, California, USA.

Wiggins DA, Schnell GD, Augustine DJ. 2014. Distribution and nesting success of ferruginous hawks and Swainson's hawks on an agricultural landscape in the Great Plains. Southwestern Naturalist **59**:356-363.

Williams BK. 1997. Logic and science in wildlife biology. Journal of Wildlife Management **61**:1007-1015.

Yasukawa K, Searcy WA. 2020. Red-winged Blackbird (*Agelaius phoeniceus*), version 1.0 in Rodewald PG, editor. Birds of the World, Cornell Lab of Ornithology, Ithaca, New York, USA. Available from <https://doi-org.ezproxy2.library.colostate.edu/10.2173/bow.rewbla.01> (accesed July 2020).

Yosef R. 2020. Loggerhead Shrike (*Lanius ludovicianus*) in Poole AF, and Gill FB, editors. Birds of the World Cornell Lab of Ornithology, Ithaca, New York, USA. Available from <https://doi-org.ezproxy2.library.colostate.edu/10.2173/bow.logshr.01> (accessed July 2020).
